# Impact of exercise training on gut microbiome imbalance in obese individuals: a study based on Mendelian randomization analysis

**DOI:** 10.3389/fphys.2023.1264931

**Published:** 2024-01-03

**Authors:** Haonan Qian, Yuxin Zuo, Shixiong Wen, Xilong Wang, Yaowen Liu, Tianwei Li

**Affiliations:** ^1^ Department of Physical Education, Hanyang University, Seoul, Republic of Korea; ^2^ Department of Health and Physical Education, The Education University of Hong Kong, Tai Po, Hong Kong SAR, China; ^3^ The University of Edinburgh, Physical Activity for Health Research Center, Edinburgh, United Kingdom

**Keywords:** gut microbiome, exercise, microbial community, causality, Mendelian randomization

## Abstract

**Objective:** The aim of this study was to investigate the relationship between exercise and gut Microbiome and to assess its possible causality.

**Methods:** Using Mendelian randomization (MR) research methods, we collected genetic data from different populations, including genetic variants associated with relative abundance or presence of microbial taxa as instrumental variables. At the same time, we extracted results related to obesity and gut Microbiome from existing relevant studies and used inverse variance weighting (IVW), weighted median, and MR-Egger regression to assess the causal relationship between obesity and gut Microbiome. We plotted forest plots and scatter plots of the association between obesity and gut Microbiome.

**Results:** Gut Microbiome was positively associated with obesity, and four bacterial genera (Akkermansia, RuminococcaceaeUCG011, Holdemania, and Intestinimonas) were associated with obesity according to inverse variance-weighted estimation in at least one MR method. Inverse variance weighted estimation showed that obesity was associated with obesity in Akkermansia (OR = 0.810, 95% CI 0.608–1.079, *p* = 0.04), RuminococcaceaeUCG011 (OR = 1.238, 95% CI 0. 511–2.999, *p* = 0.04), Holdemania Intestinimonas (OR = 1.214, 95% CI 1.002–1.470, *p* = 0.03), and Intestinimonas (OR = 0.747, 95% CI 0.514–1.086, *p* = 0.01) had a relevant effect. Obesity decreased the abundance of Akkermansia, Intestinimonas microbiome and increased the abundance of RuminococcaceaeUCG011, Holdemania microbiome.

**Conclusion:** The results of this study, conducted using a two-sample Mendelian randomization method, suggest a causal relationship between obesity and intestinal microbiome. Obesity decreased the abundance of Akkermansia, Intestinimonas microbiome and increased the abundance of RuminococcaceaeUCG011, Holdemania microbiome. More randomized controlled trials are necessary to elucidate the protective effects of exercise on gut Microbiome and its unique protective mechanisms.

## 1 Introduction

Physical activity is an important lifestyle that has a positive impact on physical health. Through active participation in physical activity, we can improve cardiovascular health, build muscle strength, increase metabolic function, and gain many other benefits. Physical activity is beneficial to the cardiovascular system. Aerobic exercise, such as running, swimming and cycling, improves heart function and blood circulation and reduces the risk of cardiovascular disease (Carmody and Bisanz, 2023; Van Hul and Cani, 2023). Moderate aerobic exercise lowers blood pressure, improves blood lipid levels, and increases the endurance of the heart. BONE HEALTH: Physical activity is essential for bone health. Gravity-loaded activities such as running, jumping and weight lifting promote increased bone density and reduce the risk of osteoporosis (Campbell et al., 2021; Lulla et al., 2022). In addition, physical activity helps improve balance and coordination, reducing the risk of falls and fractures. Physical activity builds muscle strength and flexibility. Through strength training, such as weight lifting and gymnastics, muscle mass can be increased, metabolic rate can be increased, and body shape can be improved (Arnoriaga-Rodríguez et al., 2021). Meanwhile, stretching increases muscle flexibility and joint range of motion, reducing muscle and joint discomfort. OTHER BENEFITS: Physical activity is associated with many other benefits. It can help control weight and reduce the risk of chronic diseases such as diabetes and certain cancers. In addition, physical activity improves sleep quality, increases energy levels, and promotes brain function and cognitive performance (Liu et al., 2019).

The association of physical activity on gut Microbiome has been the subject of much research attention in recent years. Gut Microbiome is the community of microorganisms that live in the human intestinal tract and contains a large number of microorganisms such as bacteria, fungi and viruses. They play an important role in human health and immune function (Aron-Wisnewsky et al., 2020). And physical exercise, as a lifestyle, has a positive impact on the composition and function of the intestinal microbiome. Studies have shown that physical activity promotes the diversity of the intestinal microbiome. Diversity refers to the number and proportion of different species of microorganisms in the microbiome (Barton et al., 2017; Carbajo-Pescador et al., 2018). Through exercise, we can improve the intestinal environment by increasing the number of beneficial bacteria and reducing the growth of harmful bacteria. This increase in beneficial bacteria helps maintain a state of balance in the gut, enhances immune system function, and reduces the risk of inflammatory diseases (De et al., 2021). In addition, physical activity increases the metabolic activity of the gut Microbiome. Studies have found that exercise can alter the production of metabolites of the gut Microbiome, such as short-chain fatty acids (SCFA). SCFA are produced by gut Microbiome fermenting dietary fiber and are essential for gut health. They provide energy to intestinal cells, maintain the integrity of the intestinal mucosal barrier (Fiuza-Luces et al., 2018), and have anti-inflammatory and anti-tumor effects. Physical activity can increase the amount of SCFA produced by the gut Microbiome after exercise, thus further promoting gut health (Cheng et al., 2022).

The association of intestinal microbiome for obesity is one of the hotspots of extensive research in recent years. Gut Microbiome refers to the community of microorganisms, including bacteria, fungi and viruses, present in the human gut. They live together with the human body and play an important role in human health and metabolic processes (Zhou et al., 2022). Several studies have shown that there is a close relationship between the composition and function of gut Microbiome and obesity. On the one hand, there may be differences in the gut Microbiome of obese individuals compared to non-obese individuals. For example, the species and abundance of certain bacteria may be significantly increased or decreased in obese individuals (Clarke et al., 2014; Seke Etet et al., 2023). These changes may be associated with energy metabolism, appetite regulation, and fat storage. On the other hand, gut Microbiome can influence the body’s digestion and absorption of food as well as energy utilization and storage, which can have an impact on body weight and body fat content. Studies have shown that gut Microbiome can be involved in regulating the onset and development of obesity through a variety of mechanisms (Morita et al., 2023). For example, certain strains of bacteria can break down indigestible dietary cellulose to produce short-chain fatty acids, which can influence appetite, energy intake and fat synthesis. In addition, gut Microbiome can influence obesity by affecting intestinal barrier function, hormone secretion and immune system regulation. However, the causal relationship between gut Microbiome and obesity has not been fully elucidated (Ray, 2014; Lai et al., 2021; Yang et al., 2023). Current research is mainly based on observational studies and animal experiments, and therefore it is not possible to determine whether changes in gut Microbiome are a cause or a consequence of obesity. In order to gain a deeper understanding of this association, researchers have conducted studies using methods such as Mendelian randomization and intestinal microbiome transplantation to investigate the causal effects of intestinal microbiome on obesity (Martínez-López et al., 2022).

In recent years, Mendelian randomization (MR) methods have been widely used to assess potential causal relationships between diseases and risk factors. Several studies have shown a correlation between exercise training and gut Microbiome, however, the causal relationship between exercise training and gut Microbiome is unclear (Wei et al., 2022). Therefore, in this study, we utilized the MR method to explore the causal association between exercise training and gut Microbiome and to provide genetic support to further understand the effects of exercise training and gut Microbiome. Through the MR approach, we utilized genetic variation as an instrumental variable to assess the effects of exercise training and gut Microbiome and to explore the causal relationship between the two (Liu et al., 2021). This approach helps to address confounders and reverse causation, which are common in observational studies, and provides more reliable evidence to support or rule out a causal association between exercise training and gut Microbiome. However, it is important to note that there are a number of limitations and assumptions associated with the MR approach, including the validity of genetic variation on the relationship between exposure and disease, the reliability of the genetic instrumentation, and the potential for incompleteness and bias, among other factors. Therefore, when performing MR analysis, we need to carefully consider these factors and combine them with other research methods and evidence to draw more accurate conclusions (Liu et al., 2021; Sales and Reimer, 2022). In summary, this study analyzed the causal relationship between exercise training and gut Microbiome using MR methods and provided genetic support. This study contributes to an in-depth understanding of the effects of exercise training and intestinal microbiome, and provides an important guideline and theoretical basis for further research on the correlation between exercise training and intestinal microbiome.

## 2 Materials and methods

### 2.1 Study design

In this study, we used Mendelian randomization (MR) to analyze the causal relationship between gut Microbiome and obesity using genetic instrumental variables. We referred to the human gut microbial genetic data published by Kurilshikov et al., in 2021 in the U.S. Department of Health and Human Services (HHS). We screened for statistically significant single nucleotide polymorphism (SNP) loci associated with human gut Microbiome as instrumental variables. With the help of these screened SNP loci, we used different MR methods to determine the causal relationship between exercise training and gut Microbiome.

### 2.2 Data sources

Exposure data for this study were obtained from a European GWAS on obesity. Based on published studies on mitochondrial DNA and sex chromosome variability in populations, we assumed that the included cases had the same genetic pattern as other patients in European countries. A total of 222,470 blood specimens from obese and 237,906 healthy controls, for a total of 460,376 study subjects, were included in the study, and 9,851,867 SNP loci were meta-analyzed by GWAS. SNP loci with *p* < 5 × 10^(−8) were selected as instrumental variables by restricting the *p*-value and chain disequilibrium conditions [*p* < 5 × 10^(−8), R^2 < 0.001, kb = 10,000]. These loci replaced intestinal microbiome as clinical risk exposure factors. The strength of the correlation between the loci and the exposure factors was judged based on the F value [F = (β/SE)^2] of each SNP, and when F > 10, it was generally considered that there was no instrumental variable bias, and SNP loci with F ≤ 10 were screened out.

### 2.3 Concluding data

MiBioGen collected 16S rRNA gene sequencing profiles and genotyping data from 18,340 subjects from a total of 11 countries in Asia and Europe, and performed locus analysis of microbiome signature loci to identify genetic loci that influence the relative abundance or presence of microbial taxa.

### 2.4 Data analysis methods

We used the TwoSampleMR package in R version 4.1.3 software for statistical analysis. The main methods included MR method: the relationship between gut Microbiome and obesity was analyzed by inverse variance weighting (IVW). IVW means of SNP ratio estimates were derived by regressing the relationship between SNP-obesity and SNP- gut Microbiome. In addition, we used weighted median estimation (WME) and MR-Egger regression as supplemental analyses. WME reduces bias in the estimation of causal effects using a weighted empirical distribution function of all study-wide SNP ratio estimates. MR-Egger regression reduces bias in the estimation of causal effects by regressing the association between SNP-obesity and SNP- gut Microbiome effect estimates through weighted linear regression regression, which reduces bias in causal effect estimates and provides valid causal effect estimates even when all SNPs are null instruments. Relatively stable causal associations can be considered to exist when the results of the three methods are in the same direction. Sensitivity analysis: Heterogeneity was detected using Cochran’s Q test and horizontal multiplicity was detected using Egger-intercept test. Results are expressed as OR (odds ratio) and 95% CI (confidence interval), with *p* < 0.05 considered statistically significant.

## 3 Results

### 3.1 Selection of instrumental variables

Sports were analyzed using 1,231 SNPs as IVs according to IV selection criteria. No weak instrumental bias was observed as the F-statistic of each SNP exceeded 10. Finally, we used 15, 13, 13, and 15 SNPs as instrumental variables for Akkermansia, RuminococcaceaeUCG011, Holdemania, and Intestinimonas bacteria, respectively, to explore the potential causal relationship between gut Microbiome and obesity.

### 3.2 Relationship between exercise and gut microbiome

Table 1 shows the four bacterial genera associated with obesity in at least one MR method, namely, Akkermansia, RuminococcaceaeUCG011, Holdemania, and Intestinimonas. Inverse variance weighted estimation showed that obesity had a significant effect on Akkermansia (OR = 0.810, 95% CI 0.608–1.079, *p* = 0.04), RuminococcaceaeUCG011 (OR = 1.238, 95% CI 0.511–2.999, *p* = 0.04), Holdemania Intestinimonas (OR = 1. 214, 95% CI 1.002–1.470, *p* = 0.03), and Enterobacteriaceae (OR = 0.747, 95% CI 0.514–1.086, *p* = 0.01) had a correlation effect. The results of Cochran’s Q-test indicated that there was no significant heterogeneity among these independent variables. In addition, MR-Egger regression intercept analysis (Additional file 1 Supplementary Table S4) showed that there was no significant directionality in multidirectionality. In addition, we detected aberrant SNPs using the MR-PRESSO technique and investigated whether the exclusion of these aberrant SNPs would alter the causal effect. Ultimately, the results of the study showed that obesity decreased the abundance of Akkermansia, Intestinimonas microbiome and increased the abundance of RuminococcaceaeUCG011, Holdemania microbiome.

**TABLE 1:** Mendelian randomization of obesity by gut Microbiome.

Exposure	MR method	SNP	F	Or (or_95%)	se	*p-*val
Akkermansia	MR Egger	15	76.23	0.722 (0.480–1.084)	0.18	0.18
	Inverse variance weighted	15		0.810 (0.608–1.079)	0.14	0.04
	Weighted mode	15		0.762 (0.537–1.081)	0.29	0.44
RuminococcaceaeUCG011	MR Egger	13	45.67	6.406 (0.041–1009.025)	0.19	0.01
	Inverse variance weighted	13		1.238 (0.511–2.999)	0.16	0.04
	Weighted mode	13		2.416 (0.421–13.851)	0.32	0.10
Holdemania	MR Egger	13	73.49	0.897 (0.476–1.690)	0.20	0.01
	Inverse variance weighted	13		1.214 (1.002–1.470)	0.16	0.03
	Weighted mode	13		1.178 (0.921–1.506)	0.30	0.09
Intestinimonas	MR Egger	15	33.41	0.745 (0.264–2.101)	0.15	0.14
	Inverse variance weighted	15		0.747 (0.514–1.086)	0.13	0.01
	Weighted mode	15		0.746 (0.467–1.191)	0.23	0.28

### 3.3 Sensitivity analysis

**FIGURE 1:**
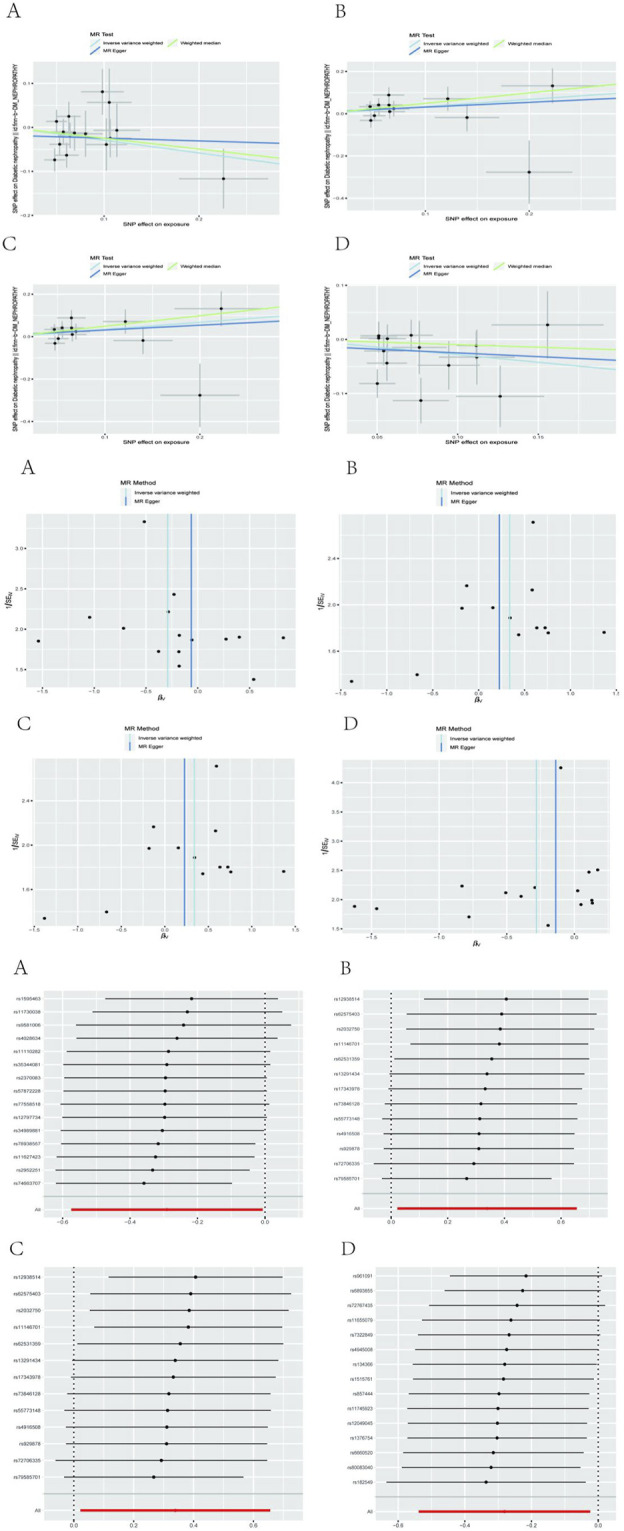
Sensitivity testing for a model of microbiome association with obesity.

After performing Cochran’s Q test, no significant heterogeneity was found among the IVs (*p* > 0.05). The MR-egger regression intercept term test showed that multiplicity did not bias the results (*p* > 0.05). The results of the analysis of pleiotropy and heterogeneity in all bacteria suggest that there was no pleiotropy and heterogeneity (*p* > 0.05; Table 2), indicating reliable results. In addition, this study used the Leave-one-out method to analyze the effect of individual SNPs on the overall results, and the results showed that the MR results were robust. Scatter diagram, funnel diagram, leave-one-out diagram (Figure 1).

**TABLE 2:** A test of multisense and heterogeneity of intestinal microbiome on obesity.

	Pleiotropy test			Heterogeneity test					
	MR-Egger			MR-Egger			Inverse variance weighted		
	Intercept	SE	*p*	Q	Q_df	Q_pvalval	Q	Q_df	Q_pvalval
Akkermansia	−0.124	0.191	0.583	3.615	5	0.606	4.228	6	0.646
RuminococcaceaeUCG011	0.001	0.078	0.996	2.482	2	0.289	3.005	3	0.391
Holdemania	0.513	0.522	0.506	NA	NA	NA	0.060	1	0.806
Intestinimonas	0.027	0.035	0.469	0.779	1	0.377	1.744	2	0.418

## 4 Discussion

In this study, we assessed the potential relationship between exercise and intestinal microbiome by MR method and found that exercise decreased the abundance of Akkermansia, Intestinimonas microbiome and increased the abundance of RuminococcaceaeUCG011, Holdemania microbiome.

There is increasing evidence that exercise training has a positive effect on the improvement of gut Microbiome in obese individuals. In this paper, we will explore the mechanism of action of exercise training to improve intestinal microbiome disorders in obese individuals. Exercise training can promote the diversity of gut Microbiome in obese individuals (Zhang et al., 2013). The increase in diversity helps to maintain the stability and function of the intestinal microbiome, reduce the growth of harmful bacteria and increase the number of beneficial bacteria, thus improving the overall balance of the microbiome. Regulates metabolic activity, exercise can alter the production of metabolites in the gut Microbiome (Zhou et al., 2021), such as short-chain fatty acids (SCFAs), which are important for intestinal health and metabolism, providing energy, regulating appetite, and promoting the function of the intestinal mucosal barrier, thereby reducing the risk of obesity. Modulation of the immune system, The improvement of gut Microbiome in obese individuals by exercise training may involve modulation of the immune system. Gut Microbiome is closely related to the immune system and influences immune function through interaction. It has been found that exercise can regulate the interaction between the intestinal microbiome and the immune system, enhance the immune function, reduce the inflammatory response, and thus improve the imbalance of the intestinal microbiome (Wang et al., 2023). In summary, the improvement effect of exercise training on gut Microbiome in obese individuals is multifaceted. It increases microbiome diversity, regulates metabolic activity, modulates immune system function as well as improves intestinal permeability. These mechanisms of action are intertwined and work together to promote the balance and health of the intestinal microbiome. However, the specific mechanisms of action need to be further investigated and may vary between individuals; therefore, personalized exercise regimens remain important for improving gut Microbiome disorders in obese individuals (Zhou et al., 2020).

Akkermansia and Intestinimonas are two common genera of gut bacteria, and their abundance has been correlated with exercise and obesity. In addition, increases in two genera, RuminococcaceaeUCG011 and Holdemania, were also associated with exercise and obesity. Akkermansia is a common genus of enterobacteria and is thought to have a high correlation with obesity (Wegierska et al., 2022). Studies have shown that Akkermansia abundance is typically lower in obese individuals. Exercise is considered an important means of improving obesity, and at the same time, exercise can increase the abundance of Akkermansia. This may be related to the fact that exercise improves the intestinal environment, increases intestinal mucosal barrier function, and modulates the immune system. Intestinimonas is another common genus of gut bacteria, and its abundance in obese individuals is also often affected. Studies have found that obese individuals typically have lower abundance of Intestinimonas (Xia et al., 2021). However, exercise workouts can significantly increase the abundance of Intestinimonas, helping to improve the imbalance of gut Microbiome. This may be due to the ability of exercise to increase the redox state of the intestinal environment, creating conditions favorable for the growth of Intestinimonas. RuminococcaceaeUCG011, a bacterial group of the Ruminococcaceae family, showed an association between increased abundance and exercise and obesity. It was shown that sports exercise significantly increased the abundance of RuminococcaceaeUCG011. Furthermore, an increase in RuminococcaceaeUCG011 was associated with a lower risk of obesity. Holdemania, a rare genus of intestinal bacteria, showed a correlation between an increase in its abundance and physical activity exercise and obesity. It was found that exercise exercise significantly increased Holdemania abundance, which was typically lower in obese individuals. This may be related to the fact that exercise workouts increase the diversity of microbiome by improving the intestinal environment and increasing the microbiome.

The results of the present study using a two-sample Mendelian randomization method, were based on inverse variance weighted estimation of a total of four bacterial genera, i.e., Akkermansia, RuminococcaceaeUCG011, Holdemania, and Intestinimonas, which were associated with exercise in at least one of the MR methods. Inverse variance weighted estimation showed that motility had a significant effect on Akkermansia (OR = 0.810, 95% CI 0.608–1.079, *p* = 0.04), RuminococcaceaeUCG011 (OR = 1.238, 95% CI 0.511–2.999, *p* = 0.04), Holdemania Intestinimonas (OR = 1.214, 95% CI 1.002–1.470, *p* = 0.03), and Intestinimonas (OR = 0.747, 95% CI 0.514–1.086, *p* = 0.01) had a correlative effect. Exercise decreased the abundance of Akkermansia, Intestinimonas microbiome and increased the abundance of RuminococcaceaeUCG011, Holdemania microbiome. It indicates a causal relationship between exercise and intestinal microbiome. Exercise decreased the abundance of Akkermansia, Intestinimonas microbiome and increased the abundance of RuminococcaceaeUCG011, Holdemania microbiome. More randomized controlled trials are necessary to elucidate the protective effect of exercise with gut Microbiome and its unique protective mechanism.

## 5 Conclusion

This study, employing a two-sample Mendelian randomization (MR) design, reveals the impact of exercise on the abundance of gut Microbiome, particularly in the genera Akkermansia, Intestinimonas, RuminococcaceaeUCG011, and Holdemania. Our findings indicate a significant association between obesity and changes in the abundance of these Microbiome, as evidenced by the associations with Akkermansia (OR = 0.810, 95% CI 0.608–1.079, *p* = 0.04) and RuminococcaceaeUCG011 (OR = 1.238, 95% CI 0.511–2.999, *p* = 0.04). These results underscore the potential role of exercise in modulating the balance of gut Microbiome. Future research should further explore the relationship between exercise and gut Microbiome through clinical studies and animal experiments, as well as validate our MR findings.

## Data Availability

The original contributions presented in the study are included in the article/Supplementary Material, further inquiries can be directed to the corresponding author.
